# Perspectives on connecting climate change and health

**DOI:** 10.1177/14034948241269748

**Published:** 2024-08-26

**Authors:** Kristin Aunan, Hans Orru, Henrik Sjödin

**Affiliations:** 1 CICERO Center for International Climate Research, Oslo, Norway; 2 Institute of Family Medicine and Public Health, University of Tartu, Tartu, Estonia; 3 Department of Public Health and Clinical Medicine, Umeå University, Umeå, Sweden

**Keywords:** Climate change, health impact assessment, transdisciplinary research, the ENBEL project

## Abstract

Over the past century, the Earth’s climate has undergone rapid and unprecedented changes, manifested in a noticeable increase in average global temperature. This has led to shifts in precipitation patterns, increased frequency of extreme weather events (e.g. hurricanes, heatwaves, droughts and floods), alterations in ecosystems, and rising sea levels, impacting both natural environments and human societies, health and wellbeing. Without deep and urgent emission cuts and effective adaptation, the toll of climate change on human health and wellbeing is likely to grow. Here, we address the complex relationship between climate change and health, and discuss ways forward for transdisciplinary research and collaboration that can motivate more ambitious mitigation policies and help develop solutions to adapt to the crisis.

## Climate affects health directly and indirectly

The global temperature is rising. In the past decade, the average global temperature has been more than 1.1°C higher than pre-industrial times, and land areas have experienced an increase of 1.6°C. It is now well established that global warming is primarily due to emissions from the combustion of fossil fuels (coal, oil and gas) and other human-driven activities, including industrial processes and deforestation [1].

According to the World Health Organization (WHO), global climate change is the greatest health risk facing humanity in the 21st century [2]. The effects of climate change on health are diverse. Some are quite direct and easily observable, while others are more indirect and challenging to assess. Examples of direct and well-established effects are those associated with extreme weather events that are now occurring more frequently, last longer, and are more intense. Recent examples of extreme events include heatwaves across Europe and Canada in the summer of 2022, with record temperatures of 40⁰C in London, the flooding in Pakistan that submerged a third of the country, flash floods in the Libyan city of Derna and devastating wildfires in Hawaii in 2023. These and similar weather events in recent years would have been highly unlikely or would not have occurred so frequently without global warming [1].

In addition to the direct health effects associated with extreme weather events, there are numerous indirect effects that can be linked to weather and climate. Climate change can increase air pollution by way of a multitude of mechanisms, resulting in enhanced health effects [3]. Another example is that rising temperatures and increased heat stress can reduce worker productivity, especially for individuals engaged in strenuous physical labour, thus having economic implications. This is particularly relevant in hot regions with low levels of mechanisation in agriculture and poorly regulated labour markets; however, recent fatalities have also been reported in France, in the Champagne region [4–6]. Other examples include effects on the environment and ecosystems that have implications for food production and access to clean drinking water. Moreover, the incidence and geographical spread of vector-borne and waterborne diseases as well as zoonoses have increased [7]. In fact, the perturbation of whole ecosystems by anthropogenic climate change can lead to shifts in the range distribution across a massive number of species, potentially triggering novel transmission routes of known and unknown pathogens, amplifying spillover frequencies and pandemic risk as well as the wider spread of allergies [8]. Extreme marine heatwaves, as seen in 2023, are linked to mass mortality events of marine organisms that might affect food availability [9].

According to the Intergovernmental Panel on Climate Change (IPCC), up to 70% of annual global deaths are attributed to diseases and conditions that are climate-sensitive, accounting for nearly 40 million deaths globally [7]. This means that mortality associated with many common causes of death could be influenced by various climate change-related phenomena, and climate change may exacerbate existing climate and weather-related health effects. It also implies that climate change could undermine the positive trends seen in global health, such as, for example, the overall reduced prevalence of stunting and wasting in low and middle-income countries [10]. Studies attributing changes in health risks to climate change are still relatively scarce, partly because there are usually multiple and complex causes of these changes. Regarding infectious diseases, for instance, even though climate change affects ecosystems and the distribution and abundance of organisms carrying zoonotic and vector-borne diseases, other factors such as land-use changes, food security, disease outbreak monitoring and control, socioeconomic conditions and general access to healthcare also play a role. While climate change affects the entire world, the health consequences are not random, and lower-income countries (e.g. in the Global South), which historically have had the lowest emissions, are often also the least equipped and prepared to adapt to climate change. Here we generally find the most vulnerable communities. Enhanced vulnerability and health impacts in poor communities can again affect economic productivity and incomes, perpetuating a cycle of poverty and further vulnerability. In its sixth assessment report (AR6), the IPCC asserts for the first time that climate change is increasingly causing involuntary migration and displacement, and may contribute to violent conflicts as well as causing disease outbreaks among climate refugees [11].

## Breaking down silos and fostering transdisciplinary collaboration

The understanding of health risks associated with climate change is growing rapidly and covering a widening range of health outcomes. The extent to which people’s health is affected depends not only on the climate hazards as such but on a range of determinants that can either enhance or mitigate the risks and health burden. Transdisciplinary collaboration is essential to understand the complex relationships between climate change and health, and to develop effective strategies for mitigating risks and enhancing preparedness. For instance, epidemiologists, biostatisticians and public health experts play a vital role in uncovering current and past connections between climate and health outcomes, including the identification of individual and contextual vulnerability factors. Social scientists and scholars in the humanities contribute by examining the behavioural, cultural and societal factors that influence vulnerability and resilience in the face of climate-related health challenges. Demography is needed to understand population dynamics, which are critical for estimating the scale of future health effects in world regions. Geographers add spatial understanding to public health challenges. Health effects entail direct costs for individuals and businesses as well as socioeconomic consequences with potentially cascading impacts within and across economies, for which economists can provide an understanding of the links. Earth systems models, developed and operated by climate scientists, are growing steadily more advanced. To harness the potential of these models fully, there is a need for collaboration with the above-mentioned disciplines to ensure that indices, temporal and spatial resolution levels and scenarios align with the requirements of other research communities addressing health impacts. Depending on the topic, co-designing studies and interventions with local communities and regional, national or international actors can help ensure policy relevance and social acceptance and support. Finally, communication and dissemination play a vital role in transdisciplinary research on climate change and health, for example, by promoting effective communication strategies empowering individuals and communities to address the challenges posed by climate change.

As has been highlighted, climate change is a worldwide phenomenon that directly or indirectly affects all countries, including Northern Europe, posing significant health consequences [12–14]. Despite commitments to both mitigating and adapting to climate change in Scandinavia, scepticism about climate change persists among the Nordic public. Thus, there is a pressing need to enhance national awareness, preparedness and resilience regarding the causes and health implications of climate change.

**Figure 1.**
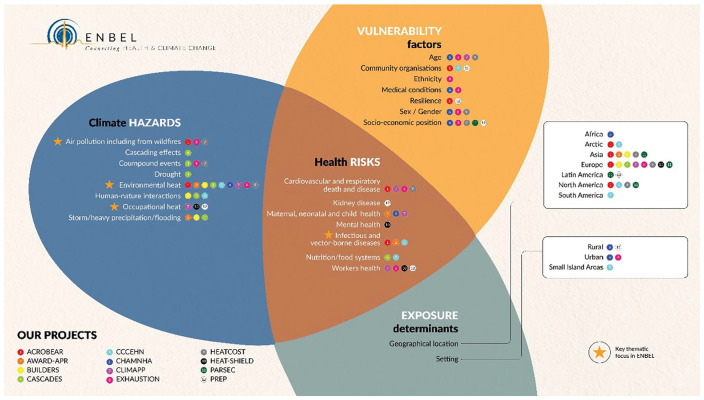
Scoping figure for the ENBEL project. The figure illustrates the climate hazards, vulnerability factors, exposure determinants and associated health risks addressed by the consortium. The figure also lists the 12 projects included and highlights their key thematic focus.

The ENBEL project (2020–2023), a cooperation and support action funded by the EU Horizon 2020 programme, brought together a network of partners involved in major international research projects on climate change and health (Figure 1). One of the core objectives has been to encourage and facilitate networking and cooperation across the often separate worlds of climate and health research communities. In the autumn of 2023, the project arranged a 2-day conference on connecting health and climate change in Stockholm, gathering more than 300 researchers, practitioners, policy advisors and other stakeholders from 35 countries around the world. Prior to this, a policy conference was held in Brussels to discuss the evidence on the health impacts of climate change and the opportunities to take action and enhance the European adaptation agenda.

In addition to providing space for sharing evidence, discussing knowledge gaps and reflecting on policy implications, several key takeaways precipitating from the conferences and the collaborative network behind them could be identified. An overarching conclusion was that the scale and urgency of climate change for health are critical and call for strengthening capacity, funding and cross-sectoral cooperation in public health research and practice. Systematic use of science is needed to design and test adaptation policies that also address the most vulnerable and hard to reach groups. Unfortunately, environmental and health data enabling research that helps to understand and prevent health risks is lacking in many regions of the world. Moreover, even if data and knowledge are available, there is a need to strengthen legal frameworks and policies to provide guidelines regarding the integration of health in climate policies. This is needed not least to ensure that adaptation and mitigation action go hand in hand, and co-benefits across health and climate measures are harvested.

## Setting global targets and tracking progress

The United Nations Framework Convention on Climate Change (UNFCCC) with its annual Conference of the Parties (COP) and the Paris Agreement on the reduction of greenhouse gas emissions and adaptation to climate change have been central to international cooperation on the climate crisis. At recent COPs, the health sector, spearheaded by the WHO, has made significant efforts to raise awareness about the health effects of climate change. The IPCC plays a crucial role in providing the scientific background for decisions made at the COPs, including those related to the Global Stocktake. We are confident that the rapidly growing climate change and health research communities will continue to provide evidence relevant to these global processes, but suggest that communities addressing climate change, impact of biodiversity and habitat loss and human health could be even better connected. Efforts such as the Global Burden of Disease Study (GBD) and the Lancet Countdown on Health and Climate Change collaboration, which track the progress of a range of indicators relevant to climate change and health (GBD estimates sub-optimal temperatures, i.e. mortality associated with high and low temperature), play an important role in providing evidence on what is at stake for human health worldwide, and how governments are delivering on commitments made under the Paris Agreement [10, 12–14]. Putting health at the core of climate action could motivate more ambitious climate policies in the critical decades ahead of us.
